# Country Almost Cleared of Salable Horses

**Published:** 1900-05

**Authors:** 


### SELECTION.

### COUNTRY ALMOST CLEARED OF SALABLE HORSES.

Horses of every class have increased from 125 to 150 per cent. in value in the last year, and judging from present indications they will continue to increase in value for the next two or three years. The same is true of mules, and despite the assertions of bicycle and automobile manufacturers, that these machines are displacing horses, the horse dealers, by statistics they have collected, show that they are not.

The facts in the matter were made public yesterday by dealers and agents who have returned to the city from the various parts of the country where they went to attend the annual spring sales of horses and mules. The causes they give for the increase in prices are as follows: First, the large purchases of horses and mules for use in the Philippines, Cuba, and Porto Rico by the United States Government. Second, the drain upon the supply caused by the large shipments to South Africa. Third, that horses are again popular among the richer class of persons in the country. Fourth, that there has been a great revival of interest in trotting and running racing.

The sales of horses this spring were double those of last May. It was also said that the United States and England had purchased 187,500 horses and mules in Missouri, Texas, Kentucky, and Tennessee and the other horse-breeding States during the past three years, at an average price of $115 each. This would make a total of $21,462,500, the largest amount ever paid for horses and mules in the same time in the history of the country.

Except in thoroughbred running horses and animals that are not suitable for military purposes, these States have practically been cleaned out of salable animals.

The agents of the British Government are now operating in the Northwest, Maine, Vermont, and the other Eastern States. They have collected 2700 more horses, which will be shipped from New York and Montreal next month. For these animals it was said an average of $135 was paid, and about a thousand more will go from Baltimore soon, and the agents are trying to get as many as possible of the 35,000 horses still wanted, through Philadelphia dealers. More than $6,000,000 was offered for this number of animals, but the dealers say that horses have become so scarce that unless the British agents are willing to accept the less desirable range animals from Wyoming, Montana, and the other Western States, the demand cannot be filled.

English agents have shipped a total of 12,875 horses and mules from New Orleans, 4000 mules and horses from Charleston, 3500 from Galveston, and also several thousand from New York and Montreal. The exact number, however, cannot now be told, as statistics have not yet been made. The United States is still purchasing horses for the Philippines, which are being shipped by way of San Francisco and Vancouver to Manila. The sales to erstwhile bicycle enthusiasts are said to have doubled.

Two or three years ago cab, light draught, and ordinary carriage horses sold for from $40 to $100 each. Trotters, saddle horses, coach horses, and medium draught horses sold from $80 to $125. Thoroughbred trotters, saddle horses, and other fine animals, when they could be sold at all, brought only from $100 to $500, except in long pedigree strains. The prices are double and treble these figures to-day. Cab, carriage, light draught, saddle, and trotting horses have become scarce, because they are most needed for cavalry, mounted infantry, and field artillery. Coach and medium and heavy draught animals have been put into the artillery, army transportation, and in the hospital corps. The mules have gone into the army transportation service with the draught horses.

Most of those from Kentucky, Missouri, and Texas are now packing loads on their backs or dragging wagons through the mud in the Philippines or among the kopjes of South Africa.

The Shetland and other ponies and the small but wiry bronchos of some of the Western States seem to be the only class of animals that are worth only a little more than they were before the bicycle craze broke out and before the two wars were begun.

As nearly five years will be required to replace the horses that have already been sent to foreign countries, and even longer to replace those that are going during the next year, horse breeding has begun to boom. It is said that hundreds of thousands of dollars have been reinvested in stock farms during the present spring and that the breeders all over the country have resumed operations, which were stopped three years ago.—*Philadelphia Times.*

